# Evaluation of the Validity of Job Exposure Matrix for Psychosocial Factors at Work

**DOI:** 10.1371/journal.pone.0108987

**Published:** 2014-09-30

**Authors:** Svetlana Solovieva, Tiina Pensola, Johanna Kausto, Rahman Shiri, Markku Heliövaara, Alex Burdorf, Kirsti Husgafvel-Pursiainen, Eira Viikari-Juntura

**Affiliations:** 1 Centre of Expertise for Health and Work Ability, Finnish Institute of Occupational Health, Helsinki, Finland; 2 Disability Prevention Centre, Finnish Institute of Occupational Health, Helsinki, Finland; 3 National Institute for Health and Welfare, Helsinki, Finland; 4 Erasmus MC, University Medical Center Rotterdam, Rotterdam, The Netherlands; University of Pennsylvania, United States of America

## Abstract

**Objective:**

To study the performance of a developed job exposure matrix (JEM) for the assessment of psychosocial factors at work in terms of accuracy, possible misclassification bias and predictive ability to detect known associations with depression and low back pain (LBP).

**Materials and Methods:**

We utilized two large population surveys (the Health 2000 Study and the Finnish Work and Health Surveys), one to construct the JEM and another to test matrix performance. In the first study, information on job demands, job control, monotonous work and social support at work was collected via face-to-face interviews. Job strain was operationalized based on job demands and job control using quadrant approach. In the second study, the sensitivity and specificity were estimated applying a Bayesian approach. The magnitude of misclassification error was examined by calculating the biased odds ratios as a function of the sensitivity and specificity of the JEM and fixed true prevalence and odds ratios. Finally, we adjusted for misclassification error the observed associations between JEM measures and selected health outcomes.

**Results:**

The matrix showed a good accuracy for job control and job strain, while its performance for other exposures was relatively low. Without correction for exposure misclassification, the JEM was able to detect the association between job strain and depression in men and between monotonous work and LBP in both genders.

**Conclusions:**

Our results suggest that JEM more accurately identifies occupations with low control and high strain than those with high demands or low social support. Overall, the present JEM is a useful source of job-level psychosocial exposures in epidemiological studies lacking individual-level exposure information. Furthermore, we showed the applicability of a Bayesian approach in the evaluation of the performance of the JEM in a situation where, in practice, no gold standard of exposure assessment exists.

## Introduction

During the past three decades, the effects of psychosocial factors at work on health have received considerable attention in research. Psychosocial factors at work are numerous, with psychological job demands, job control (decision latitude), efforts and rewards [1], [2] comprising the key dimensions. Another factor of importance is social support at work [3].

The job strain model, introduced by Karasek in 1979 [4], is one of the most studied occupational stress models. According to the model, workers with a combination of high psychosocial job demands and low control over a job (high job strain) have a higher risk of developing an illness as compared to workers with low psychosocial job demands and high job control (low job strain) [1]. The job strain model has been successfully used to predict the risk of cardiovascular disease [5], [6], major mental disorders [7], type II diabetes [8] and musculoskeletal diseases [9]. The effects of the individual components of the job strain model on health have also been evaluated, although the results have often been inconsistent across the studies and health outcomes [7], [9].

The interpretation of the observed associations between psychosocial factors at work and health mainly depends on the validity of the assessment methods of the risk factors. Self-reported questionnaires are widely used to measure psychosocial factors at work [10]. Self-reports provide subjective information representing a worker's perception of occupational stress and are therefore susceptible to reporting bias. The subjective assessment of psychosocial factors at work has been the largest concern in the debate on the interpretation of associations and on the possible causal role of these factors for illness. It has been suggested that common source bias due to subjective measures of psychosocial factors at work increases the likelihood of false positive findings, particularly in cross-sectional studies with the self-reported health outcomes [11]–[13]. Workers having health problems are more likely to report certain psychosocial exposures than healthy workers. Such tendency might lead to differential misclassification, which results either in an overestimation or underestimation of the true effect [14], particularly, when exposures and outcome are measured simultaneously.

The assessment of psychosocial factors at work with a job exposure matrix (JEM), where exposure level is assigned based on the job-specific average of exposure, is not prone to information bias and may therefore guarantee some degree of objectivity. The major advantage of the JEM in epidemiological studies is that it can be applied to the populations with lacking exposure information. However, such method of exposure assessment induces Berkson type error, which may not cause notable bias on the effect estimates but weakens the precision of the estimates [15]. A JEM neglects both within worker (over time variation) and between worker (variation in tasks, activities and work processes) variation in a job [16] and therefore may result in false positive and negative exposure assignments for a considerable proportion of the subjects. A non-differential misclassification bias induced by JEM will attenuate the observed associations towards null [15], [17], [18]. Knowing the magnitude of measurement error (e.g. sensitivity and specificity) and exposure prevalence, the extent of non-differential bias can be estimated [15], [19].

Several psychosocial job exposure matrices have been developed and used in epidemiological studies [20]–[26]. Even though the JEM measures are more objective than self-reported ones, they cannot be seen as a gold standard in the context of psychosocial factors at work [13]. Therefore, the question of the reliability of the associations between JEM-based exposures and health outcomes is always warranted. The validity of psychosocial JEM measures in the absence of a gold standard method is challenging to evaluate and, as a result, has rarely been examined and reported [24]–[28]. Furthermore, the magnitude of misclassification bias of psychosocial JEM measures on effect estimates has not been examined so far.

The aims of the study were 1) to examine the accuracy of a developed gender-specific job exposure matrix in the assessment of psychosocial factors at work applying the Bayesian approach, 2) to evaluate the theoretical impact of exposure misclassification on exposure-outcome associations and 3) to examine the ability of the matrix to detect known associations between psychosocial factors at work and health outcomes.

## Materials and Methods

### Study population

We utilized two large Finnish population samples. The Health 2000 (H2000) Study was used to construct the JEM and to examine the inter-method agreement, and the national Finnish Work and Health (FWH) Surveys were used to test the performance of the matrix. The study populations consisted of 18–64 year-old individuals, who had been working during the preceding 12 months.

The Health 2000 Study is a large Finnish population-based study carried out in 2000–01. The main objective of the study was to obtain representative information on the current health status of the whole non-institutional adult population in Finland. The survey consisted of several questionnaires, a home interview, and a health examination. A nationally representative sample of the population was obtained using a two-stage stratified cluster sampling design. The original samples consisted of 8028 subjects aged 30 years or over and 1894 subjects aged 18–29. The participation rates were 87% and 90%, respectively. A detailed comprehensive description of the methods and processes has been published elsewhere [29], [30]. The sample of this study comprised 4619 persons aged 18–64 who were working during the preceding 12 months and for whom information on occupational titles and exposures were available. The age and gender distribution of the study population matched those of the employed persons in Finland in the year 2000.

The national Finnish National Work and Health Surveys have been conducted every third year since 1997 to collect information on perceived working conditions and the health of the working-age population, For the 1997–2003 Surveys, random samples of subjects aged 25–64 years independent of their working status (e.g., working, unemployed, retired or student) were drawn from the Finnish population register. For the 2009 Survey a random sample of subjects aged 20–64 years was drawn from Finnish employment statistics. The sample size has varied between 2031 and 2355 persons from year to year with a response rate of 58–72% [31]. At each survey, a phone number was not found for about 10–16% of subjects. The proportion of non-participants in each survey was slightly higher among men than women and among subjects aged 24–34 years than among the older subjects. Age, gender, education, socioeconomic status and occupational sector of the respondents were compared with the Census data. No major differences were found. Thus, the respondents to the FWH Surveys represent rather well the targeted population. The data from all five surveys were combined. Hence, the total number of the interviewed persons with information on occupation during 1997–2009 was 11326.

The H2000 Study and the FWH Surveys have all obtained ethical approval from the appropriate ethics committees.

### Classification of occupations

Occupations in both surveys were classified on the 4-digit level (including few occupations coded with 5 digits) according to the Classification of Occupations 2001 by Statistics Finland, which is based on the International Standard Classification of Occupations (ISCO-88). The classification is based on ten categories of professional skills. In total, the classification includes 444 job titles.

### Psychosocial exposures

Psychosocial exposures in the H2000 Study were measured with a Finnish version of the Job Content Questionnaire (JCQ) [32]. The JCQ has been shown to be a valid and reliable instrument to assess job stress and social support in many occupational settings worldwide [10], [13]. Responses were given on a five point Likert-scale from 1 (fully agree) to 5 (fully disagree).

Psychological *job demands* scale is the sum of the following five items: “work fast”, “work hard”, “excessive work”, “not enough time”, and “hectic job”. In the current study, Cronbach's alpha for the scale was 0.76 for men and 0.81 for women. *Job control* scale is the sum of two subscales. Decision authority was measured with three items: “allows own decisions”, “decision freedom”, and “a lot of say on the job”), and skill discretion was measured with five items: “learn new things”, “requires creativity”, “high skill level”, “variety”, and “develop own abilities”. Cronbach's alpha for the scale was 0.85 for men and 0.86 for women. Since *monotonous (repetitive) work* was weakly correlated with the other five items of the skill discretion scale we treated it as a separate exposure. Job demands, job control and monotonous work were dichotomized using gender-specific median cut-off points.

*Job strain* was operationalized using the quadrant approach proposed by Karasek and Theorell [1]. It defines workers who are above the median on job demands and below the median on job control as having a high strain job. Other categories are: low strain (low demands and high control), passive (low demands and low control) and active (high demands and high control). Low strain job was used as the reference category in the analyses.

Social *support* at work was measured with four items: “support from supervisor”, “supervisor appreciates”, “support from co-workers”, “discussion on work”. Cronbach's alpha for the scale was 0.80 for men and 0.82 for women. Social support was dichotomized at a gender-specific median in order to define low and high support.

### Development of the job exposure matrix (JEM)

We constructed a gender-specific matrix with exposure estimates at each intersection between rows (occupational groups) and columns (psychosocial exposures). The exposure axis of the matrix included the above mentioned five psychosocial risk factors at work. The occupation axis of the matrix was based on the original job titles or occupational groups.

Previous studies showed that ten individuals with the same job title will be sufficient for a reliable estimation of exposures [33], [34]. The exposure estimates for job demands, job control, monotonous work and social support at work were calculated as a median score of exposures in each occupation which included at least 10 subjects in order to obtain reasonably precise estimates. The exposure estimates for job strain were calculated as the proportion of exposed to passive, active and high strain work. The job titles with a small number (<10) of respondents were grouped based on the similarities of these job titles with regard to work tasks (including supervising), work environment, and required educational level. The gender differences in the exposures were also considered. If there was no reasonable way to merge the occupation with other occupations within the gender (such as female frontier guards), the exposure estimates of both genders in that occupation/occupational group were combined.

The sample size of the H2000 Study was large enough to enable us to develop a gender-specific job exposure matrix and to keep several job titles unmerged. Out of 444 possible job titles, altogether 363 (300 among men and 267 among women) were available in the Health 2000 Study. There were 61 job titles among men and 58 among women with at least 10 subjects. These job titles covered 69% of the study sample. After merging the smaller groups the number of job titles or occupational groups reduced to 110 among men and 101 among women.

The exposure estimates for job demands, job control, monotonous work and social support at work were dichotomized using gender-specific median as a cut-off point. The categories of job strain were obtained based on the dichotomized JEM-based job demands and job control.

### Health outcomes

Based on the current evidence we chose two health outcomes that are known to be associated with psychosocial factors at work. Both cross-sectional and longitudinal studies have shown that high level of psychological demands and job strain are associated with major mental disorders [7], [35]–[37]. Suggestive evidence for a relationship of job demands, job control and monotonous work with low back pain has also been reported [9], [38], [39].

#### Depressive symptoms

In both studies, depressive symptoms were assessed with the following question: “Have you had melancholy or depression during the last month (30 days)?”. The response categories ranged from 1 = not at all to 5 = very often. The occurrence of depressive symptoms was dichotomized as no (categories 1 and 2) or yes (categories from 3 to 5).

#### Low back pain

In the H2000 Study information on low back pain was inquired with the following question: “Have you had pain in your back during the past month (30 days)?” (yes/no). In the FWH Surveys, data on low back pain were collected with an interview using the question: “Have you during the past month (30 days) had long-lasting or recurrent pain in the lumbar spine?” (yes/no).

### Data analyses

In the H2000 Study, the inter-method agreement between self-reported and JEM measures was examined using intra-class correlation (ICC). Two-way mixed total ICC agreements were computed. In the FWH Surveys, the performance of the matrix was evaluated by examining the accuracy of the matrix in the identification of exposed/non-exposed individuals, estimating exposure misclassification error, and looking at the ability of the matrix to detect associations of psychosocial factors at work with one-month prevalence of depression or low back pain (predictive validity) [40]. The accuracy of the JEM was evaluated using five indicators: sensitivity (Se), specificity (Sp), Youden's J index, likelihood ratio positive (LR+) and likelihood ratio negative (LR−).

Sensitivity (ability of the test to identify positive results) and specificity (ability of the test to identify negative results) are usually determined against a reference standard test (gold standard). Errors in measuring the sensitivity and specificity of a test will arise if the reference test itself does not have 100% sensitivity and 100% specificity. Since there is no gold standard measure for psychosocial factors at work, we estimated sensitivity and specificity using a Bayesian approach, proposed by Joseph et al. [41]. As the first step, the posterior distribution of sensitivity and specificity of the JEM measures was calculated using self-reported and JEM measures of exposures from H2000 Study. For these analyses, the prior distribution of the parameters was derived based on the assumption that the self-reported measures have almost perfect sensitivity and specificity and no prior information on sensitivity and specificity of JEM measures is available. As the second step, the posterior distribution of sensitivity and specificity of the JEM measures was calculated using data from FWH Surveys. For these analyses the prior distributions of the parameters were derived based on the posterior distributions obtained in the first step. At each step, the posterior medians and their 95% Bayesian credible intervals were estimated using Gibbs sampler algorithm with WinBUGS software version 1.4.3.

The estimated sensitivity and specificity were used to calculate Youden's J index as well as LR+ and LR−. The Youden's J index (J = Se+Sp−1) has been used as a measure of the effectiveness of the JEM to discriminate between exposed and non-exposed individuals. The possible range of the Youden's J index value is between 0 (totally useless) and 1 (perfect). Likelihood ratio positive is the probability of an exposed person to be classified as exposed divided by the probability of a non-exposed person to be classified as exposed. Likelihood ratio negative is the probability of an exposed person to be classified as non-exposed divided by the probability of a non-exposed person to be classified as non-exposed. A likelihood ratio equal to 1 will indicate that the JEM measure is unable to distinguish between exposed and non-exposed. A LR>1 will indicate that the JEM is likely to identify exposed and LR<1 will indicate that the JEM is likely to identify non-exposed. The higher LR+ value and lower the LR− value, the better is the JEM performance.

To estimate the theoretical magnitude of exposure misclassification, biased odds ratios (OR′) were calculated based on the obtained estimates of sensitivity (Se) and specificity (Sp) and assumed “true prevalence” (Pr) and “true odds ratios” (OR) using the following formula [19]:
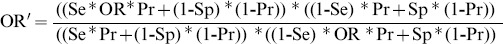



The true prevalence was fixed at 0.50 for high job demands, low job control and low social support, at 0.33 for monotonous work and at 0.25 for high strain job. The true odds ratios were fixed at three values OR = 1.5, OR = 2 and OR = 3. The relative difference between biased and true estimates was calculated ((OR′-OR)/OR) and used as quantitative measure for the magnitude of exposure misclassification.

Logistic regression analyses with age, education and year of survey (the FWH Surveys) adjusted odds ratios (OR) and 95% confidence intervals (CIs) were carried out to study the associations between the JEM measures and one-month prevalence of depression or low back pain. These analyses were performed using SAS version 9.1. The effect estimates were adjusted for misclassification error using WINPEPI COMPARE2 program, version 3.08 [42].

All analyses were performed separately for men and women.

## Results

In both genders, the prevalence of high job demands, high job strain and monotonous work measured by job exposure matrix was statistically significantly lower than that assessed by self-reports (Table 1). In women, the prevalence of low social support was lower for JEM measures than for self-reported measures. There were no differences in the distribution of exposures assessed by JEM between the two study populations, reflecting a similar job distribution in both surveys. In general, total agreement between self-reported and JEM measures assessed by ICC was slightly better among women than men, with the largest ICC values observed for job control followed by monotonous work (Table 2).

**Table 1:** Prevalence of self-reported and JEM-based psychosocial exposures.

	Men			Women		
Exposures	H2000 Study		FWH Surveys	H2000 Study		FWH Surveys
	Self-reported	JEM	JEM	Self-reported	JEM	JEM
	Prev. (95% CI)	Prev. (95% CI)	Prev. (95% CI)	Prev. (95% CI)	Prev. (95% CI)	Prev. (95% CI)
High job demands	43.1 (41.1, 45.0)	33.1 (29.4, 33.3)	32.2 (31.0, 33.4)	44.2 (42.2, 46.3)	35.3 (33.4, 37.3)	36.0 (34.7, 37.3)
Low job control	52.6 (50.5, 54.7)	49.8 (47.8, 51.9)	49.8 (48.5, 51.1)	56.1 (54.1, 58.1)	56.5 (54.5, 58.5)	55.4 (54.1, 56.7)
Job strain						
Low strain job	26.2 (24.4, 28.1)	36.3 (34.3, 38.3)	36.3 (34.1, 36.6)	23.8 (22.1, 25.6)	24.6 (22.9, 26.4)	25.6 (24.4, 26.3)
Passive job	30.7 (28.8, 32.7)	32.4 (30.5, 34.4)	32.5 (31.3, 33.8)	32.0 (30.1, 33.9)	40.0 (38.1, 42.1)	38.4 (37.2, 39.7)
Active job	21.2 (19.5, 22.9)	13.9 (12.5, 15.4)	14.9 (14.0, 15.8)	20.0 (18.4, 21.7)	18.9 (17.3, 20.5)	19.0 (18.0, 20.1)
High strain job	21.9 (20.2, 23.6)	17.4 (15.9, 19.1)	17.3 (16.3, 18.3)	24.2 (22.5, 26.0)	16.4 (15.0, 18.0)	16.9 (16.0, 18.0)
Low social support	47.3 (45.3, 49.4)	48.5 (46.4, 50.1)	48.4 (47.1, 49.7)	44.2 (42.2, 46.2)	37.1 (35.2, 39.1)	37.5 (36.2, 38.7)
Monotonous work	28.5 (26.6, 30.4)	17.2 (15.7, 18.8)	17.1 (16.1, 18.1)	31.8 (29.9, 33.7)	24.0 (22.3, 25.8)	22.1 (21.0, 23.2)

H2000 Study- the Health 2000 Study; FWH Surveys- the Finnish Work and Health Surveys.

**Table 2:** Intra-class correlation (ICC) between individual-based and group-based measures of psychosocial exposures for men and women in the H2000 Study.

	Men	Women
Job demands	0.31	0.40
Job control	0.53	0.60
Monotonous work	0.45	0.51
Social support	0.36	0.33
Job strain	0.21	0.29

H2000 Study- the Health 2000 Study.

### Bayesian estimates of sensitivity and specificity and magnitude of exposure misclassification error

The Bayesian estimates of sensitivity and specificity were calculated based on the data from the Health 2000 Study and The Finnish Work and Health Surveys and are shown in the form of posterior medians and 95% Bayesian intervals (Table 3). The posterior estimates were very similar in both study populations. The specificity of JEM measures was higher than sensitivity for all exposures except job control among women. Specificity ranged from 0.62 to 0.90 in men, and from 0.68 to 0.86 in women. Sensitivity was the lowest for high strain job (0.46) in men and for low social support (0.52) in women. The best matrix performance assessed by Youden's J index and likelihood ratios was found for high strain job, particularly in women. The JEM was least effective in identification of men exposed to high demands (J = 0.17) and women exposed to low social support (J = 0.15).

**Table 3:** Posterior medians and lower and upper limits of the posterior equally tailed 95% credible intervals (Bayesian confidence interval).

		H2000 Study		FWH Surveys				
		Sensitivity	Specificity	Sensitivity	Specificity	J^1^	LR+^2^	LR−3
**High demands**	Men	0.41 (0.38–0.44)	0.76 (0.74–0.78)	0.41 (0.39–0.44)	0.76 (0.74–0.78)	0.17	1.71 (1.53–1.91)	0.78 (0.73–0.82)
	Women	0.49 (0.46–0.52)	0.75 (0.73–0.78)	0.49 (0.46–0.52)	0.75 (0.73–0.78)	0.24	2.00 (1.77–2.25)	0.68 (0.63–0.72)
**Low control**	Men	0.67 (0.64–0.70)	0.69 (0.67–0.72)	0.67 (0.64–0.69)	0.70 (0.67–0.72)	0.37	2.20 (2.01–2.42)	0.48 (0.43–0.52)
	Women	0.75 (0.73–0.78)	0.68 (0.65–0.71)	0.76 (0.73–0.78)	0.68 (0.65–0.71)	0.44	2.35 (2.14–2.59)	0.36 (0.32–0.40)
**High strain job**	Men	0.58 (0.52–0.64)	0.87 (0.84–0.91)	0.60 (0.55–0.65)	0.82 (0.77–0.86)	0.42	3.34 (2.52–4.45)	0.49 (0.42–0.57)
	Women	0.77 (0.71–0.82)	0.88 (0.84–0.92)	0.78 (0.73–0.83)	0.86 (0.81–0.90)	0.64	5.75 (4.13–8.03)	0.25 (0.19–0.31)
**Low social support**	Men	0.60 (0.57–0.63)	0.62 (0.59–0.65)	0.60 (0.57–0.63)	0.62 (0.59–0.65)	0.22	1.59 (1.45–1.74)	0.65 (0.59–0.71)
	Women	0.48 (0.45–0.51)	0.72 (0.70–0.74)	0.44 (0.42–0.46)	0.71 (0.69–0.73)	0.15	1.52 (1.40–1.65)	0.79 (0.75–0.83)
**Monotonous work**	Men	0.35 (0.32–0.39)	0.90 (0.88–0.91)	0.36 (0.32–0.39)	0.90 (0.88–0.91)	0.26	3.39 (2.84–4.01)	0.72 (0.68–0.76)
	Women	0.46 (0.42–0.50)	0.86 (0.84–0.88)	0.46 (0.42–0.50)	0.86 (0.84–0.88)	0.32	3.31 (2.79–3.91)	0.63 (0.58–0.68)

The sensitivity and specificity of JEM-based measures for men and women in the H2000 Study and the FWH Surveys.

H2000 Study- the Health 2000 Study; FWH Surveys- the Finnish Work and Health Surveys;

1 J - Youden's index = sensitivity+specificity−1.

2 LR+ likelihood ratio positive.

3 LR− likelihood ratio negative.

The theoretical effect of exposure misclassification error on estimated ORs is shown in Table 4. In both genders, the smallest misclassification error was observed for high job strain, followed by that for low job control. The largest misclassification error was found for low social support (both genders) and high job demands (men). In general, when the true OR is equal to 1.5, the effect of misclassification error on point estimates is relatively small, though there is a high likelihood of false negative findings. A statistically significant association can be detected only for low job control and high job strain in women. With the increase of true OR, there is a larger reduction in the biased odds ratios, but at the same time the likelihood of false negative findings is lowered.

**Table 4:** Biased odds (OR′) ratios according to sensitivity and specificity of the job exposure matrix when the true odds ratios (OR) were assumed to equal 1.5, 2 or 3.

	OR = 1.5		OR = 2.0		OR = 3.0	
	Men	Women	Men	Women	Men	Women
High job demands^1^	1.08	1.11	1.13	1.18*	1.21*	1.28*
Low job control^1^	1.16	1.20*	1.28*	1.35*	1.45*	1.58*
Monotonous work^2^	1.17	1.17	1.30*	1.31*	1.50*	1.52*
Low social support^1^	1.09	1.07	1.16	1.11	1.25*	1.17*
High job strain^3^	1.18	1.27*	1.34*	1.53*	1.60*	1.99*

1 Prevalence of exposure is assumed to equal 0.50.

2 Prevalence of exposure is assumed to equal 0.33.

3 Prevalence of exposure is assumed to equal 0.25.

*Statistical significance at the 5% level (two-sided test) of the biased odds ratios is calculated for a study population of 5000 men and 5000 women.

### Predictive validity of the JEM measures

The one-month prevalence of depression was statistically significantly higher in the H2000 Study as compared with the FWH Surveys, while the prevalence of low back pain during the preceding 30 days was similar (Table 5). In both study populations, women tended to report depression and LBP more frequently than men.

**Table 5:** The association of psychosocial exposures measured at individual (ind) level and at group level (job exposure matrix (JEM)) with one-month prevalence of depression and low back pain among men and women.

		High demands		Low control		Monotonous work		Low social support		High strain job	
	Prevalence (95% CI)	OR	95% CI	OR	95% CI	OR	95% CI	OR	95% CI	OR	95% CI
**Men**											
***Depressive symptoms***											
H2000 (ind)^1^	13.9 (12.3–15.5)	1.98	1.51–2.59	1.52	1.16–2.01	1.45	1.07–1.95	2.07	1.59–2.74	3.07	2.05–4.62
FWH (JEM)^2^	10.6 (9.8–11.5)	1.15	0.96–1.37	1.19	0.98–1.43	0.95	0.75–1.21	1.11	0.93–1.31	1.34	1.04–1.72
FWH (JEM)^3^		2.08	1.74–2.48	1.33	1.12–1.59	0.81	0.67–0.99	1.54	1.30–1.72	1.70	1.34–2.15
***Low back pain***											
H2000 (ind)^1^	26.8 (25.0–28.7)	1.19	0.99–1.44	1.13	0.93–1.37	1.01	0.81–1.26	1.16	0.96–1.41	1.37	1.04–1.80
FWH (JEM)^2^	29.8 (28.6–31.0)	1.04	0.92–1.18	1.06	0.94–1.21	1.22	1.05–1.42	1.06	0.94–1.19	1.10	0.92–1.30
FWH (JEM)^3^		1.20	1.07–1.34	1.73	1.54–1.95	2.38	2.10–2.69	1.50	1.33–1.68	1.77	1.51–2.09
**Women**											
***Depressive symptoms***											
H2000 (ind)^1^	18.7 (17.0–20.5)	1.55	1.22–1.95	1.49	1.17–1.90	1.29	1.00–1.67	2.75	2.16–3.50	2.38	1.68–3.37
FWH (JEM)^2^	15.9 (14.9–16.9)	0.99	0.85–1.15	1.07	0.91–1.28	1.01	0.84–1.22	0.97	0.83–1.13	1.09	0.87–1.37
FWH (JEM)^3^		0.88	0.76–1.01	1.33	1.15–1.54	1.24	1.05–1.45	0.79	0.69–0.92	1.20	0.96–1.50
***Low back pain***											
H2000 (ind)^1^	29.7 (27.8–31.6)	1.29	1.08–1.55	1.27	1.05–1.54	1.16	0.95–1.43	1.28	1.07–1.54	1.68	1.28–2.20
FWH (JEM)^2^	31.4 (30.2–32.)	1.00	0.89–1.13	1.12	0.99–1.27	1.19	1.03–1.38	1.00	0.89–1.12	1.14	0.95–1.38
FWH (JEM)^3^		0.92	0.82–1.03	1.76	1.56–1.97	2.38	2.10–2.71	0.83	0.74–0.94	1.52	1.27–1.81

Odds ratios (OR) and their 95% confidence intervals (95% CI).

H2000 – the Health 2000 Study; FWH- the Finnish Work and Health Surveys;

1 ORs calculated based on self-reports and adjusted for age and education.

2 ORs calculated based on JEM and adjusted for age, education and year of survey.

3 ORs calculated based on JEM adjusted for exposure misclassification bias.

In the H2000 Study, associations between all self-reported psychosocial factors at work and depression were statistically significant in both genders (Table 5). In the FWH Surveys, the point estimates of associations between the JEM-based exposures and depression were reduced by 22–65% as compared with those for self-reported exposures in the H2000 Study, particularly in women. The smallest drop was found for low job control (men) and monotonous work (women), while the largest reduction in estimates was observed for low social support in women. After correction for exposure misclassification, the odds ratios obtained with JEM regained their statistical significance for low job control (both genders), monotonous work (women), and high job demands, low social support and high strain job (men). However, women with high job demands or low social support assessed by JEM had reduced odds of depression. Similarly, monotonous work seemed to be associated with lower risk of depression in men.

All self-reported psychosocial factors at work, except monotonous work, were statistically significantly associated with LBP in women (Table 5). In men, high job demands, low job control and low social support tended to increase the odds of LBP, although the association was statistically significant for high job strain only. The estimated odds for JEM-based exposures were reduced by 6–21% in men and by 12–32% in women as compared with those for self-reported exposures. Unexpectedly, for monotonous work, the odds ratios obtained with JEM were increased by 21% as compared to odds ratios obtained with self-reports. After correction for exposure misclassification error, all JEM-based exposures in men and all except high job demands in women were statistically significantly associated with LBP. Women with low social support had a low prevalence of LBP.

## Discussion

We comprehensively validated a gender-specific job exposure matrix that we constructed for the assessment of psychosocial factors at work. The matrix showed a good accuracy in identification of individuals exposed to low job control and high job strain, while its performance for job demands and social support was relatively low. The largest misclassification error was found for low social support (women) and high job demands (men). The difference between the odds ratios based on self-reports and JEM was larger for depression than for low back pain, especially in women. Without correction for exposure misclassification, the JEM was able to detect the association between job strain and depression in men and that between monotonous work and low back pain in both genders. The predictive ability of the matrix substantially improved after correction for possible misclassification bias.

Although several psychosocial JEMs exist, their validity is poorly explored. Most of the previous studies on the validation of JEMs examined their ability to detect known associations between JEM measures and health outcomes (predictive validity) [24]–[28]. Few studies evaluated inter-method agreement between JEM and self-reported measures [24], [43]. There are several parameters that can be used to evaluate the performance of an exposure assessment method, of which sensitivity, specificity, Youden's J index and likelihood ratios are the most commonly applied. Considering all performance indicators, the performance of our JEM was good for job control and job strain and was rather low for job demands and social support. These findings are in line with the results of the previous studies that reported higher validity of the JEM measures for job control and job strain than for job demands and social support [13], [43], [44]. The relatively low validity of job demands may suggest that variation of this factor between occupations is smaller than that within occupation [20], [21]. However, the poor performance for social support may alternatively reflect that some psychosocial factors are highly individually oriented in that a particular job may be perceived as very strenuous for some whereas not for others.

Among performance indicators, sensitivity and specificity are the key ones, because all others are calculated based on them. Theoretically, sensitivity and specificity should be determined against a reference test (gold standard). In practice, the sensitivity and specificity of the JEMs are usually evaluated against self-reports, even if it is well known that the self-reported exposures may be subject to information bias. In the current study, we used the Bayesian approach to estimate sensitivity and specificity of JEM measures. The similarity of estimates obtained in both of our study samples suggests their robustness. The sensitivity of the JEM-based estimates for job control and high strain job was acceptable, while it was reduced for job demands, monotonous work and social support. The specificity of all our JEM-based estimates varied from good to very good and was substantially higher, especially in women, as compared to those found in a French study [24].

The studies that examined the predictive validity of the psychosocial JEM measures have consistently reported weaker associations between JEM measures and health outcomes than what has been found for the corresponding self-reported factors [24]–[28]. In general, the associations of JEM measures for job strain and job control with health outcomes were better reproducible than the associations for job demands. However, even unexpected results of a protective effect of high job demands assessed by JEM on anxiety disorders [25] and self-rated health [24] have been reported.

When JEM is used to study the association between an exposure and a health outcome, there is always some loss of information because the individual values are replaced with the group-based (job title) ones. Both self-reported exposures and JEM are prone to classification errors whose consequences on effect estimates need to be considered when interpreting the association between the exposure and the outcome. The measurement error in exposures assessed by JEM is always of a Berkson type, while the error of self-reported measures is of a classical type. The group-specific average of exposures used in our JEM was obtained based on nationally representative self-reported exposure data; therefore, the measurement error of our JEM has both classical and Berkson component, with the latter being dominant. The classical and Berkson errors bias the effect estimates differently [15]. The Berkson error has almost no effect on the point estimate, while it severely affects the estimate's precision. In case of classical error, the direction and magnitude of bias are more difficult to assess. We observed a larger difference between the self-reported and JEM-based exposures in the ORs for depressive symptoms than for LBP. This may suggest the presence of a higher common source bias in self-reported exposure measures among those reporting depressive symptoms than among those reporting LBP. As a result, for depressive symptoms, the risk estimates based on JEM measures may be closer to the true risk than the risk estimates based on self-reports. These benefits support the use of the JEM in epidemiological studies.

The ability of the JEM to detect known associations between risk factors and health outcomes primarily depends on the magnitude of misclassification error. Even though studies have examined the predictive validity of psychosocial JEM measures, none of them examined the effect of exposure misclassification on observed associations. Our results suggest that, due to misclassification error, we were not able to observe associations between job demands, job control and social support assessed by JEM with either depression or low back pain. However, after correction for misclassification bias, the ability of the matrix to detect the expected associations improved substantially. Furthermore, the bias-adjusted effect estimates for low job control and high job strain in our study were about the same as those reported in previous meta-analyses [7], [9].

## Conclusions

Our results suggest that JEM more accurately identifies occupations with low control and high strain than those with high demands or low social support. Although the JEM is a rather crude exposure assessment method, it can be a useful source of job-level psychosocial exposures in epidemiological studies lacking individual-level exposure. Furthermore, we showed the applicability of a Bayesian approach in the evaluation of the performance of the JEM in a situation where, in practice, no gold standard of exposure assessment exists.
